# Condition-Dependent Trade-Off Between Weapon Size and Immunity in Males of the European Earwig

**DOI:** 10.1038/s41598-017-08339-6

**Published:** 2017-08-11

**Authors:** Maximilian Körner, Fanny Vogelweith, Susanne Foitzik, Joël Meunier

**Affiliations:** 1 Institute of Organismic and Molecular Evolutionary Biology, Johannes-Gutenberg University of Mainz, Mainz, Germany; 2 Institut de Recherche sur la Biologie de l’Insecte, UMR 7261, CNRS, François-Rabelais University of Tours, Tours, France

## Abstract

Investigating the expression of trade-offs between key life-history functions is central to our understanding of how these functions evolved and are maintained. However, detecting trade-offs can be challenging due to variation in resource availability, which masks trade-offs at the population level. Here, we investigated in the European earwig *Forficula auricularia* whether (1) weapon size trades off with three key immune parameters – hemocyte concentration, phenoloxidase and prophenoloxidase activity - and whether (2) expression and strength of these trade-offs depend on male body condition (body size) and/or change after an immune challenge. Our results partially confirmed condition dependent trade-offs between weapon size and immunity in male earwigs. Specifically, we found that after an immune challenge, weapon size trades off with hemocyte concentrations in low-condition, but not in good-condition males. Contrastingly, weapon size was independent of pre-challenge hemocyte concentration. We also found no trade-off between weapon size and phenoloxidase activity, independent of body condition and immune challenge. Overall, our study reveals that trade-offs with sexual traits may weaken or disappear in good-condition individuals. Given the importance of weapon size for male reproductive success, our results highlight how low-condition individuals may employ alternative life-history investment strategies to cope with resource limitation.

## Introduction

Optimal allocation of resources into physiological, morphological and behavioural traits is typically known to determine the fitness of an individual^1^. Investing heavily into one life-history trait can, however, reduce the resources available for other processes. This trade-off is common in nature and often affects the intricate balance between survival and reproductive prowess, such as fecundity^2–4^ and male weaponry and ornaments. These latter traits are large, extravagant structures that are energetically costly to develop^5, 6^ but increase males’ reproductive success by enhancing their fighting abilities and/or appeal to female mate choice^7, 8^. Males of the stag beetle *Cyclommatus metallifer*, for example, are well known to express extremely large and conspicuous weapons that improve mating success, but come at costs in terms of wing size and flight muscles^9^.

In nature, the expression of a trade-off between mutually exclusive functions is ultimately governed by the overall available resources an individual can allocate to them^10, 11^. Any change in these resources, for instance due to environmental, physiological, and/or genetic variation, is thus expected to mask investment trade-offs or even result in an apparently positive relationship between two specific traits^11–13^. Many studies are in line with these predictions. For instance, high-quality females have been shown to exhibit diminished costs of reproduction compared to low-quality ones in two species of ungulates^14^ and males with increased access to resources show a reversed fecundity-longevity trade-off apparently lowering the cost of reproduction in the seed beetle *Callosobruchus maculatus* ^15, 16^. Conversely, several studies report a rather puzzling absence of trade-offs between sexually-selected and non-sexual traits. For example, in the rhinoceros beetle *Trypoxylus dichotomus*, large horns seem to impose no costs on overall growth, mobility, or immunity^17^, and in the horned beetle *Euoniticellus intermedius*, where parts of the immune response were shown to positively correlate with male horn length with no sign of a trade-off^18^.

The reversal or removal of trade-offs has been suggested for male sexual traits as part of the handicap principle^19^, which aims to explain how male sexual traits evolved and more importantly, why they - in theory - must be honest, i.e. reflect the condition of the bearer. In a refined model based on the handicap principle, Grafen proposed in 1990^20^ that marginal costs of advertising sexual traits are higher for individuals in poor condition. This suggests that trade-offs involving sexual traits should be less taxing on other life-history traits in high compared to low-condition males and thus result in an overall positive growth allometry in high condition males only (relative scaling of body parts)^21^. This condition-dependent effect, however, has received mixed support across species^22^, and studies investigating condition-dependence in sexual trait expression rarely include trade-offs with other, non-sexual traits^23^. Yet, condition dependency of sexually-selected weapons or ornaments in males has been established in a number of species, such as horn length in the isopod *Deto echinata* ^24^, eye span in the stalk-eyed fly *Diasemopsis aethiopica* ^25^, or weapon size in the cactus bug *Narnia femorata* ^26^. To what degree this condition dependency of sexually-selected traits affects any trade-offs with other, non-sexual traits remains largely unknown.

**Figure 1:**
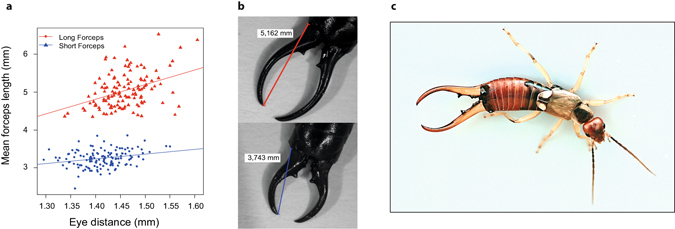
(**a**) Different association between eye distance (proxy for body size) and forceps length for long forceps males (P > 0.001) and short forceps males (P = 0.041). The difference in correlation was accounted for when calculating the relative forceps length used in the analyses. (**b**) Illustrative examples of measured long and short forceps. (**c**) Male specimen of *Forficula auricularia* with long forceps (Photo: Joël Meunier).

In this study, we investigated whether trade-offs between sexual (forceps length) and life-history (immunity) traits are condition-dependent in males of the European earwig *Forficula auricularia*. In this hemimetabolous insect with a promiscuous mating system^27^, males are well known to wield curved forceps at the end of their abdomen (Fig. 1). The length of male forceps - which varies at adulthood only - is positively associated with the duration and frequency of copulations, with their general fighting abilities, and with their capability to interrupt the mating attempts of contenders^28, 29^. Male forceps size is a heritable trait^30^ that can vary dramatically within a population^29, 31^. During initial development and continuing through adulthood, longer forceps are likely to be more costly than smaller forceps for individuals due to their encumbering size and weight^9^. Here, we tested if forceps length trades off with males’ investment into immunity. Immunity is a cornerstone of defence against pathogens and parasites but is also costly and therefore often expected to trade-off with life-history traits^32, 33^. Specifically, we investigated three key components of males’ immune system: phenoloxidase activity (PO), prophenoloxidase activity (PPO; which is measured together with PO as total-PO), and hemocyte concentration^34–36^. PO and its inactive precursor, PPO, help individuals resist a large number of pathogens through melanisation and the induction of the release of various cytotoxins^35, 37, 38^, while hemocytes are involved in core immune functions such as phagocytosis, nodulation, and encapsulation^34^. Although PO and total-PO are often expected to correlate, interesting discrepancies have been reported in the literature making the dual measurement worthwhile^39^. The investments into immune components were measured both before (basal) and 24 h after (activated) an immune challenge, since the degree and direction of the expected trade-offs are not necessarily the same during these two physiological stages^40, 41^. The immune challenge was done by pricking individuals with a sterile needle either dipped into a control solution (i.e. injury = low immune challenge) or into a lipopolysaccharide solution (LPS = high immune challenge), a component of gram-negative bacteria cell walls that is generally used as a non-pathogenic immune elicitor in insects^42–44^. If the condition of a male determines the presence and direction of an investment trade-off between weapon size and immunocompetence in the direction predicted by Grafen^20^, we expected to detect a trade-off between forceps length and the level of basal immunity and/or immune response in the smallest (i.e. low quality) but not the largest (i.e. high quality) males.

## Materials and Methods

### Insect rearing and definitions of weapon size categories

To investigate condition-dependent trade-offs between forceps length and immunity, we first selected a large number of males exhibiting strong variation in this sexually-selected trait. To this end, we field-caught 1188 males and 1296 females of *F. auricularia* in July-August 2015 from a single natural population located in Mainz, Germany (49°58′20.5″N 8°11′42.3″E). Immediately after field sampling, we distributed these individuals into 36 plastic containers (37 × 22 × 25 cm) grounded with moist sand to homogenize nutrition, habitation and access to mates for the males. Each container received 36 females and 33 males, which were allowed to freely mate for four months. After that time, the 1188 males were sorted to select the top 9% exhibiting the longest forceps and the bottom 9% with the shortest forceps. To this end, we first visually selected a large subset of long and short forceps males and then measured their mean forceps length (as the mean of left and right outer forceps) and eye distance as two proxies of body size^45^ and quality^46, 47^ (detailed selection process in supp. materials). Note that variation in earwig body size reflects both their genetic background and their previous environment^30, 48^. These measurements eventually provided us with a total of 112 “short forceps” males (i.e. the 9.4% of males with the shortest forceps) and 105 “long forceps” males (i.e. the 8.8% of males with the longest forceps) (Fig. 1). After these morphology measurements, each male was isolated in a Petri dish (5.5 cm diameter) and provided with an *ad libitum* quantity of standard food (composition detailed in Kramer *et al*.^45^) for 24 hours, before it was used for immune measurements and/or immune challenge (see below). The Petri dishes were furnished with moist sand and maintained under standard laboratory conditions^49^. All morphometric measurements were done following standard protocols^27, 47, 50^, in which sizes were taken to the nearest 0.001 mm using the Leica Application Suite 4.5 software (Leica Microsystems, Wetzlar, Germany) on pictures of CO_2_ anesthetized males taken under a binocular scope (Leica, MZ 12.5).

### Basal immunity and immune challenges

The association between weapon size and immunocompetence was investigated by measuring male investment into three components of their immune system both before (basal) and 24 hours after (activated) an immune challenge. The basal immunity was measured one day after each of the 217 short- and long-forceps males were isolated (note that social isolation does not influence male capacity to fend off pathogens and is thus unlikely to shape their immunocompetence)^51^. At that time, 1 µl of hemolymph was extracted per male (median volume of extraction = 1.0 µl; min = 0.6 µl; max = 2 µl) to measure the number of circulating hemocytes as well as total-PO and PO activities (see below). Just after hemolymph extraction, a random subset of 58 of the short-forceps males and 50 of the long-forceps males (n = 108 total) were immune-challenged by pricking them with a sterile needle that was previously dipped either 1) into a suspension of LPS (diluted in a Ringer solution at 10 mg/ml; n = 29 short- and 27 long-forceps) or 2) into a control solution (100% Ringer; n = 29 short- and 23 long-forceps). The remaining 109 non-challenged males were used in another experiment (data not shown). All these pricked males were then returned to their Petri dish, where they were given *ad libitum* access to food for 24 hours. The immune response of the 107 surviving males (one long-forceps male died) was finally determined by re-extracting 1 µl of their hemolymph (median = 1.0 µl; min = 0.4 µl; max = 1.4 µl) and measuring hemocyte concentration, as well as PO and total-PO activities (see below).

### Measurement of the three immune parameters

The 1 µl of hemolymph per individual to be used for the hemocyte, PO, and total-PO measurements was diluted in 25 µl of cold sodium cacodylate/CaCl_2_ buffer, of which 10 µl were used immediately to measure hemocyte concentration while 16 µl were frozen to later measure PO and total-PO. If the initial amount of hemolymph was less than 1 µl, we noted the actual amount using a glass capillary and a calliper. The concentration of hemocytes was then measured by visual count using a Neubauer Improved Haemocytometer and a microscope (magnification ×400). The PO and total-PO activities were spectrophotometrically measured using a standard protocol^52^. In brief, each frozen sample of diluted hemolymph was thawed on ice and centrifuged for 5 minutes at 4 °C (4000 × g), after which 5 µl of the resulting supernatant was added to a microplate well containing 20 µl of PBS, 20 µl of L-dopa solution (Sigma D-9628; 4 mg/ml of distilled water), and either 140 µl of distilled water (PO activity) or 140 µl of chymotrypsin solution (Sigma C-7762, 0.07 mg/ml of distilled water; total-PO activity). The enzymatic reaction was allowed to proceed for 2 hours 47 minutes at 30 °C in a microplate reader (Thermo scientific Multiskan™ FC Microplate Photometer). Enzyme activity was defined as the slope of the reaction curve during the linear phase of the reaction (Vmax value: change in absorbance units/min) and measured using the R-based free program PO-CALC^53^. Because the volume of extracted hemolymph and the resulting concentration of hemolymph slightly varied between individuals (see the range of extraction detailed above), we standardized the concentration of hemocytes and total-PO activity (immune parameters) per microliter of hemolymph using the following formula: I x [(Vh + Vb)/Vh]/Vm, in which I is the measured immune parameter, Vh is the volume of extracted hemolymph, Vb is the volume of buffer added, and Vm is the volume applied either to the Haemocymeter for hemocyte count (i.e. 10 µl) or on the spectrophotometer plate for total-PO measurement (i.e. 5 µl).

### Statistical analyses

We first tested whether hemocyte concentration, PO activity, and/or total-PO activity depended on males’ body size and forceps length using a series of six general linear models (function *lm* in R). Three models were computed with the immune values taken before the immune challenge, whereas three other models were conducted with the immune values taken after the immune challenge. Note that in the last three models, the type of challenge (control or LPS) was also entered as an explanatory factor, and that we controlled for the values of the considered basal immune trait by entering them as covariate. Each statistical model first included all possible interactions between the explanatory factors (i.e. body size, forceps length and, when available, the type of challenge) and was then simplified stepwise by removing the interaction terms that were not significant (all P-values > 0.08) after which we confirmed best model selection using Akaike Information Criterion (AIC). Note that some non-significant interactions are presented here to facilitate model comparisons, but their removal from the statistical models did not qualitatively change the results. In all six models, forceps length was corrected for body size within each male category, as these two values are positively associated, but the slope of this association depends on the male size category (Fig. 1). This correction was done by using the residuals of two general linear models (one for the long- and one for the short-forceps males), in which the forceps length was entered as a response variable and the body size as an explanatory variable. This corrected forceps length thus provided information on whether males had longer or shorter forceps than predicted by their body size within each forceps category, which is exactly the focus of the present study.

To fulfil homoscedasticity and Gaussian distribution of the residuals, all the models were computed using square root-transformed hemocyte concentration and log +0.001-transformed PO and total-PO activities. The statistical analyses were conducted using R v3.1.2 loaded with the packages *car* and *effects*. This latter package was used to plot and interpret the interactions between continuous variables, as it displays the predicted relationship between the response variable and one explanatory variable for different, fixed values of the interacting variable(s) (see refs 54 and 55).

### Data availability

The complete dataset is archived in Dryad. Doi:10.5061/dryad.q03d6.

## Results

Prior to the immune challenge, there was no trade-off between forceps length and the immune defence of males. Specifically, the baseline concentration of hemocytes and the baseline activities of PO and total-PO were independent of male body size (hemocytes: *F*_1,206_ = 1.99, *P* = 0.16; PO: *F*_1,196_ = 0.41, *P* = 0.525; total-PO: *F*_1,205_ = 0.49, *P* = 0.484), as well as of forceps length (hemocytes: *F*_1,206_ = 2.69, *P* = 0.103; PO: *F*_1,196_ = 1.6, *P* = 0.207; total-PO: *F*_1,205_ = 0.01, *P* = 0.914) and of an interaction between these two traits (all *P* > 0.196).

**Figure 2:**
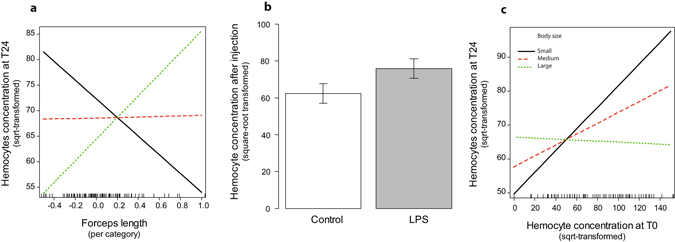
Effects of (**a**) the interaction between body size and the forceps length on hemocyte concentration 24 h after the immune challenge, (**b**) the type of immune challenge, and (**c**) the interaction between body size and basal hemocyte concentration. In the first and third panel, the straight lines represent males with small body size (1^st^ quartile of the distribution = 1.407), where the dashed lines represent the males with medium body size (median value = 1.437), and the dotted lines represent males with large body size (3^rd^ quartile of the distribution = 1.47). The dashes on the abscissa represent the distribution of males.

**Figure 3:**
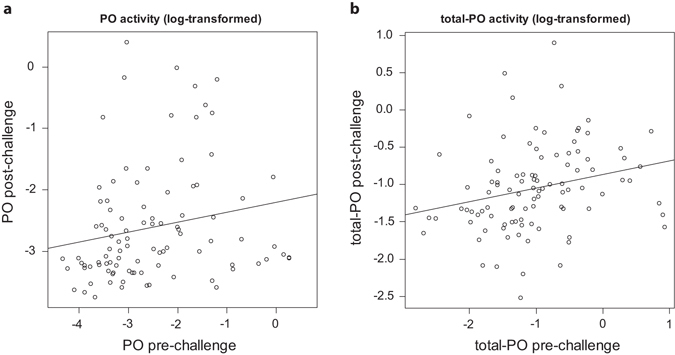
Association between the measurements of (**a**) PO and (**b**) total-PO activities before and after the immune challenge.

Conversely, after the immune challenge, there was a trade-off between forceps length and hemocyte concentration, but its expression depended on males’ body size (interaction between body size and forceps length, *P* = 0.009; Table 1). Specifically, hemocyte concentration traded off with forceps length in the smaller males, whereas hemocyte concentration increased together with forceps length in the largest males (Fig. 2a). Independent of this effect, post-challenge hemocyte concentration was overall higher in LPS-pricked than in control-pricked males (*P* = 0.015, Table 1 and Fig. 2b) and the post-challenge and basal levels of hemocyte concentrations were positively correlated in the smaller males only (interaction between body size and basal measurements, *P* = 0.004; Table 1 and Fig. 2c). Hemocyte concentrations were, however, not shaped by an interaction between the type of immune challenge and body size (Table 1). Finally, post-challenge activities of PO and total-PO were overall positively correlated to their baseline activities (all *P* < 0.025; Fig. 3 and Table 1), but were independent of forceps length, body size, and the type of immune challenge (all *P* > 0.231; Table 1). Note that all measures of PO and total-PO activities were independent of hemocyte concentrations (all *P* > 0.086; Table 2).

**Table 1:** Effects of basal immunity, body size, forceps length, type of pricking (control versus LPS), and their interaction on the hemocyte concentration, PO, and total-PO activities after the injections of LPS or control solutions.

	Hemocyte number		PO activity		Total-PO activity	
	F_(1,98)_	P	F_(1,89)_	P	F_(1,92)_	P
Basal measurement (Bm)	3.98	**0.049**	5.17	**0.025**	7.07	**0.009**
Body size (BS)	3.16	0.078	0.00	0.951	1.45	0.231
Forceps length (FL)	1.05	0.309	1.34	0.251	0.14	0.709
Type of pricking	6.16	**0.015**	0.14	0.712	0.07	0.795
Body size: Basal measurement	8.49	**0.004**	0.45	0.502	1.01	0.317
Body size: Forceps length	7.21	**0.009**	0.14	0.711	0.18	0.668

The basal immunity corresponds to the value of each immune parameter measured 24 h before the pricking. Significant P-values are in bold. These three models first included all possible interactions between the explanatory factors and were then simplified (see Methods).

**Table 2:** Spearman correlation tests among hemocyte concentration, PO, and total-PO activities measured (a) before and (b) after pricking. Significant P-values are in bold.

	Hemocyte concentration	PO activity	Total-PO activity
**a) Measurements before immune challenge**			
Hemocyte concentration	n = 210	rs = 0.06	rs = 0.12
PO activity	P = 0.412	n = 200	rs = 0.81
Total-PO activity	P = 0.086	**P** < **0**.**0001**	n = 209
**b) Measurements after immune challenge**			
Hemocyte concentration	n = 107	rs = −0.15	rs = −0.12
PO activity	P = 0.134	n = 101	rs = 0.67
Total-PO activity	P = 0.278	**P** < **0**.**0001**	n = 101

## Discussion

We investigated whether the forceps size of field-sampled earwig males traded off with their immunocompetence and whether the expression and direction of this trade-off depended on male body size. Our data partially confirm our predictions: we showed that male forceps size traded off with hemocyte concentration in small males after an immune challenge (independent of the type of immune challenge). In large males, however, post-challenge hemocyte concentration did not trade-off but instead increased with forceps size. Our results also reported an absence of trade-off or positive association between forceps length and the basal concentration of hemocytes, as well as between forceps length and either the basal or the post-immune challenge levels of PO and total-PO activities.

Our results show a condition-dependent trade-off between forceps size and the post-challenge concentration of hemocytes in earwig males. This result is in line with the trade-off between sexual traits and immunocompetence reported in numerous species ranging from vertebrates (barn swallows *Hirundo rustica*)^56^ to invertebrates (crickets *Gryllus campestris* ^32^; wolf spiders *Hygrolycosa rubrofasciata*)^57^. Here, however, our findings reveal that even if investment into sexual ornamentation and weaponry is a costly affair, this cost is significant (in terms of immune function) only for males exhibiting an overall small body size. Knowing the general benefits of exhibiting long forceps in earwig males’ mating success^28, 29^, these results suggest that the emergence of alternative mating strategies limiting the importance of forceps length could be favoured in small males^58^. In line with this hypothesis, male earwigs with short forceps have been shown to sneak copulations more frequently than better armed males^59^. Alternatively, small males may still opt for reduced investment into immunocompetence to increase forceps growth and therefore better compete with larger conspecifics^60, 61^ when the risks of pathogen infections are low in the population, or when high levels of competition facilitate a high-risk/high-reward mating strategy. Disentangling these different evolutionary scenarios would require further investigations into the importance of pathogen pressure on forceps development in young males, as well as their expression of alternative mating strategies. Note, however, that a study recently revealed that forceps length is highly heritable in this species^30^, suggesting that short forceps males might use an alternative mating strategy to reach a similar fitness as long forceps males.

The condition-dependent trade-off between forceps length and hemocyte concentration only appeared after an immune challenge, regardless of challenge type (pricking or control). This is somewhat surprising, since the basal number of circulating hemocytes is traditionally assumed to reflect an individual’s ability to mount an immune response^34, 62^. Nevertheless, our results may reveal that upregulating immune capacity in response to a threat could be more cost efficient than maintaining a constantly high level of immunity. This has been previously proposed in larvae of the moth *Eupoecilia ambiguella* ^40^, where body size is positively associated with hemocyte concentration after, but not before an immune challenge.

Although the LPS-pricked males showed an overall higher hemocyte concentration than control-pricked males, the type of immune challenge had no effect on PO and PPO. These results confirm that the wounding itself is sufficient to trigger an immune reaction in terms of hemocyte concentration, a phenomenon reported in several other insects^40, 63, 64^, but insufficient to trigger an upregulation of PO/PPO. This latter absence of effect was surprising, as the concentration we used is relatively high (e.g. 20× higher than what was required to elicit an immune response in the bumblebee *Bombus terrestris* ^44^, an insect with a comparable body weight). Nevertheless, it illustrates that various immunity pathways may react differently to immune challenges^34, 40, 65^.

Similarly, the condition-dependent trade-off between forceps length and immunocompetence affected the hemocyte concentration, but not the PO/PPO activity. While immune parameters do not always represent pathogen resistance equally^34, 41, 65^, past studies have reported associations of the PO/PPO enzyme cascade with pathogen response^36^ and with individual condition^66, 67^. Interpreting the immune function of high or low levels of PO/PPO activities can, however, be difficult. This is because reaction products of PO activation include several proteases and oxygen radicals that can actually harm the host as well as the intruder, meaning that an overexpression can prove costly and even detrimental through autoreactive damage^67, 68^. As a result, having a more capable, stronger immune system may not automatically imply higher cytotoxic responses, like those induced by the PO cascade. Showing that males of different size and/or quality exhibit an equal response in the PO/PPO enzyme cascade could thus actually reflect the better condition of the larger individuals, since they only have to pay for a relatively meek immune response in comparison to their smaller conspecifics.

While we did not detect any correlation between body or forceps size and baseline hemocyte concentration, we found that the association between pre- and post-challenge hemocyte concentration depended on the size of the males. In small males, the post-challenge concentration of hemocytes was higher when the basal concentration of hemocytes was also high (and vice versa). This means that small males fit our initial expectations of basal hemocytes indicating an individual’s ability to mount an immune response^34, 62^. In large males, however, the recruitment of hemocytes after an immune challenge appears to be independent of their basal concentration of hemocytes. Overall, these results are in line with the assumption that body size is a reliable indicator of individual quality^46, 47, 49, 69–71^ and hint at a condition-dependent change in immune investment.

Overall, our results reveal that being a short- or a long-forceps male (regardless of the traits that may covary with forceps length under natural conditions) has important implications in terms of immunity in male earwigs, mostly regarding hemocyte concentration. By using field-sampled individuals that may have experienced natural events specific to their forceps length, we also showed that trade-offs between crucial traits such as sexually-selected weapons/ornaments and immunocompetence can be condition-dependent within the same sex, population, and habitat. While condition dependency of sexually-selected traits has been demonstrated before, how their apparently variable costs affect and trade-off with other important traits was largely unknown. This is, however, of special importance since the cost of ornaments and therefore the associated trade-offs with other life-history traits are often thought to be crucial for the evolution of honest signalling^19, 72, 73^. Finally, our data show that sexual traits like weaponry can be of higher priority than immunocompetence in poor condition individuals, indicating a high-risk/high-reward strategy, while good condition individuals can equally invest in both traits to strike an even balance between attractiveness and survival.

## Electronic supplementary material

Supplementary Material
