# Supplementary material for: Condition-Dependent Trade-Off Between Weapon Size and Immunity in Males of the European Earwig
Source: Sci Rep. 2017 Aug 11;7:7988. doi: 10.1038/s41598-017-08339-6 (PMC5554132; doi:10.1038/s41598-017-08339-6)
Supplement: Supplementary file 1 — Supplementary Material [file 41598_2017_8339_MOESM1_ESM.pdf]

Supplementary Materia for:

CONDITION-DEPENDENT TRADE-OFF BETWEEN WEAPON SIZE AND IMMUNITY IN MALES OF THE EUROPEAN EARWIG

Maximilian Körner[1*#], Fanny Vogelweith[1*], Susanne Foitzik[1], Joël Meunier[1,2]

[1] Institute of Organismic and Molecular Evolutionary Biology, Johannes-Gutenberg University of Mainz, Mainz, Germany

[2] Institut de Recherche sur la Biologie de l'Insecte, UMR 7261, CNRS, François-Rabelais University of Tours, Tours, France

[*]Authors contributed equally to the study

# Corresponding author: Maximilian Körner

Email: maxkoerner@gmx.net

Tel.: +49 6131 39-27853

Fax: +49 6131 39-27850

S1: Selection Process

In order to properly select the longest and shortest forceps males from our pool of 1188 males, we conducted a multi-stage selection process. Males from all 36 containers were **1)** randomly distributed in three subsets of 12 containers. Each subset was then **2)** visually scanned to extract 44 to 46 males with the longest and 44 to 46 males with the shortest forceps (the number of males selected per container ranged from 3 to 11). We then **3)** measured the mean forceps length (as the mean of left and right outer forceps) and the eye distance as a proxy of both body size[1] and individual quality[2,3].

Based on these morphometric measurements, we subsequently refined the long-forceps category as a forceps length that exceed the average forceps length (4.11 mm) of the 274 males by at least 5% (min = 4.32 mm, max = 6.53 mm) and the short-forceps category as a forceps length that was by at least 5% lower than the average length (min = 2.475 mm, max = 3.9 mm). These final criteria excluded 57 of the 274 males and provided us with a total of 112 "short forceps" males (i.e. the 9.4% of males with the shortest forceps) and 105 "long forceps" males (i.e. the 8.8% of males with the longest forceps) (see main text, Figure 1). All morphometric measurements were taken to the nearest 0.001 mm using the Leica Application Suite 4.5 software (Leica Microsystems, Wetzlar, Germany) on pictures of CO_2 anesthetized males taken under a binocular scope (Leica, MZ 12.5).
